# Rapid Colorimetric Assay for Detection of *Listeria monocytogenes* in Food Samples Using LAMP Formation of DNA Concatemers and Gold Nanoparticle-DNA Probe Complex

**DOI:** 10.3389/fchem.2018.00090

**Published:** 2018-04-03

**Authors:** Sirirat Wachiralurpan, Thayat Sriyapai, Supatra Areekit, Pichapak Sriyapai, Suphitcha Augkarawaritsawong, Somchai Santiwatanakul, Kosum Chansiri

**Affiliations:** ^1^Department of Biochemistry, Faculty of Medicine, Srinakharinwirot University, Bangkok, Thailand; ^2^Faculty of Environmental Culture and Ecotourism, Srinakharinwirot University, Bangkok, Thailand; ^3^Innovative Learning Center, Srinakharinwirot University, Bangkok, Thailand; ^4^Department of Microbiology, Faculty of Science, Srinakharinwirot University, Bangkok, Thailand; ^5^Department of Pathology, Faculty of Medicine, Srinakharinwirot University, Bangkok, Thailand

**Keywords:** *L. monocytogenes*, *plcB* gene, loop-mediated isothermal amplification (LAMP), gold nanoparticle/DNA probe, rapid colorimetric detection

## Abstract

*Listeria monocytogenes* is a major foodborne pathogen of global health concern. Herein, the rapid diagnosis of *L. monocytogenes* has been achieved using loop-mediated isothermal amplification (LAMP) based on the phosphatidylcholine-phospholipase C gene (*plcB*). Colorimetric detection was then performed through the formation of DNA concatemers and a gold nanoparticle/DNA probe complex (GNP/DNA probe). The overall detection process was accomplished within approximately 1 h with no need for complicated equipment. The limits of detection for *L. monocytogenes* in the forms of purified genomic DNA and pure culture were 800 fg and 2.82 CFU mL^−1^, respectively. No cross reactions were observed from closely related bacteria species. The LAMP-GNP/DNA probe assay was applied to the detection of 200 raw chicken meat samples and compared to routine standard methods. The data revealed that the specificity, sensitivity, and accuracy were 100, 90.20, and 97.50%, respectively. The present assay was 100% in conformity with LAMP-agarose gel electrophoresis assay. Five samples that were negative by both assays appeared to have the pathogen at below the level of detection. The assay can be applied as a rapid direct screening method for *L. monocytogenes*.

## Introduction

Since foodborne pathogens can be transmitted to the human body through the consumption of contaminated food, it is of critical importance to perform early detection of pathogens in a variety of food samples (Velusamy et al., 2010). Such demands have fueled the development of innovative techniques for the precise, rapid, and user-friendly point-of-care (POC) detection of pathogens. Among them, *Listeria monocytogenes* is a food-borne pathogen that is non-spore forming, facultatively anaerobic, and exhibits tumbling motility, propelled by peritrichous flagella. It is capable of tolerating environmental stress, such as surviving under acid stress conditions as low as pH 2.5 (Ferreira et al., 2003), and recovering after treatment with 100 mM Tris at pH 12.0 (Liu et al., 2005). Therefore, *L. monocytogenes* can be difficult to control in terms of food safety. Traditional methods for the detection of this pathogen include various cultural, biochemical, and immunological methods, but these approaches are laborious and time consuming. At present, molecular biological methods play an essential role in medical diagnostics, food safety analysis, and environmental monitoring. Methods such as nucleic acid amplification are becoming increasingly popular due to being rapid, reliable, sensitive, and specific (Zhang et al., 2009, 2018; Frece et al., 2010). One of the most important and efficient molecular biology-based methods for the specific detection of pathogens is loop-mediated isothermal amplification (LAMP). The LAMP reaction is detected by measuring the fluorescent intensity of the indicated reagents (Fischbach et al., 2015) or the turbidity of the DNA polymerization by-products (magnesium pyrophosphate; Mori et al., 2001; Jayawardena et al., 2007). These methods are capable of obtaining real time signals, from which the amount of the DNA template can be quantified (Liu et al., 2017; Wachiralurpan et al., 2017b). The visual detection of turbidity by the naked eye was achieved when a fluorescent dye such as SYBR green was added to the solution after the LAMP reaction (Mashooq et al., 2016). However, the color change from orange to green is not significant enough to observe by eye under ambient light when the very small amount of target DNA was amplified. Thus, a UV irradiation box is sometimes required for better assessment.

At the present, LAMP-LFD is the membrane-based technique that has been applied for detection of various pathogens due to its convenience, rapid, and friendly to users. However, the membrane treatment should be concerned in order to minimize the background interference (Wang et al., 2006; Rodríguez et al., 2016).

**Figure 1:**
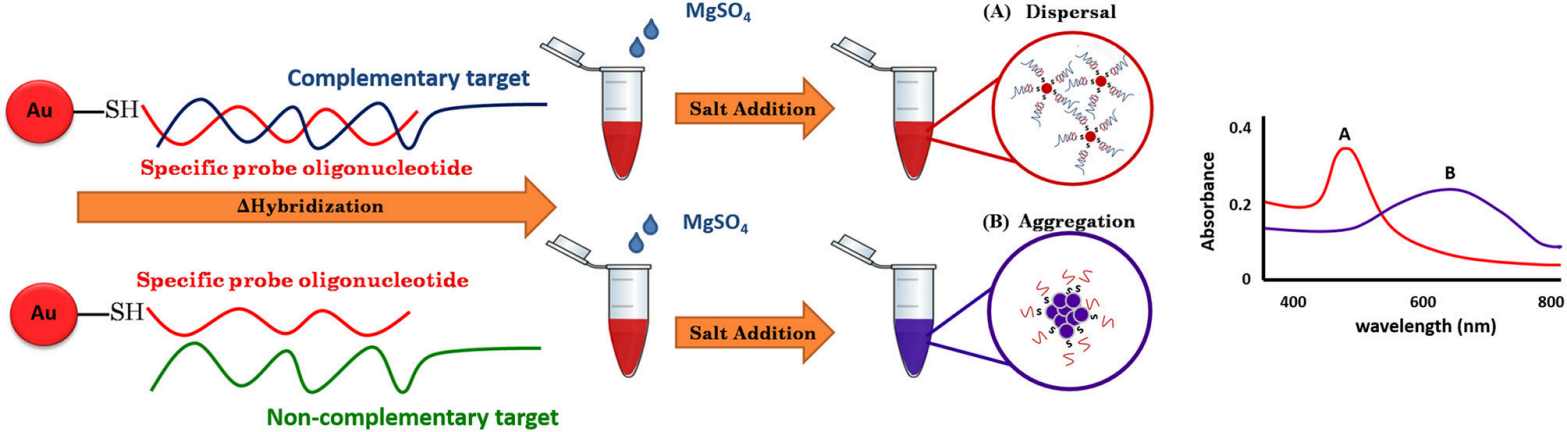
A schematic illustration of LAMP-GNP/DNA probe assay.

For the use of gold nanoparticles (GNPs) in bio-sensing, the conjugated biomolecules act as bio-receptors that recognize the target molecule and can then easily be visualized by the color change of the GNP-target complex without any expensive or complex instrumentation (Elghanian et al., 1997). The basic outline of the approach is given in Figure 1. The key to the GNP/DNA probe assay is the control of the dispersion and aggregation of colloidal GNPs by salt concentration for colorimetric visual detection. By such means, the absence of the targeted nucleic acid sequence (negative sample), upon hybridization and addition optimum salt concentration, the surface charge of GNP/DNA probe becomes neutral, causing nanoparticles to aggregates immediately, as indicated by the solution color changes from red to blue and/or gray color and the UV-vis absorption peak toward the wavelength at 550–650 nm. Conversely, in the presence of the targeted nucleic acid sequence (positive sample), the GNP/DNA probe can be complementary to the positive sample form to the hybridized complex with polymeric network for supporting the nanoparticle dispersion under ionic strength condition, and as a result, no colorimetric change and the UV-vis absorption peak could be observed at wavelength 520–550 nm that slightly shift to the original GNP/DNA probe (Baptista et al., 2008; Seetang-Nun et al., 2013).

In this study, a simple and rapid colorimetric method for the direct identification of *L. monocytogenes* has been achieved. Under appropriate conditions, the test samples with their complementary target DNA sequences hybridized with the GNP/DNA probe remained red, whereas non-complementary target DNA sequences resulted in the aggregation of GNP and a concomitant color change from red to purple and then to colorless. The GNP/DNA probe assay can be applied as a screening tool at POC or in less well-equipped basic field laboratories.

## Materials and methods

### Bacterial strains and one-step DNA extraction

Eight strains of *Listeria* species and 27 strains of non-*Listeria* bacteria were tested using LAMP and LAMP-GNP/DNA probe assays to show the specificity of the methods. All bacterial strains were prepared as laboratory stocks obtained from the culture collection (Table 1). A pure strain of *L. monocytogenes* DMST 17303 was used as the resource for culturing and preparation of genomic DNA.

**Table 1:** Bacterial isolates for LAMP-GNP/DNA probe specificity test.

**Bacterial isolate**	**Source**
***Listeria monocytogenes*** (***n*** = **5**)	
*L. monocytogenes* DMST 17303	DMST
*L. monocytogenes* DMST 20093	DMST
*L. monocytogenes* DMST 21164	DMST
*L. monocytogenes* DMST 23145	DMST
*L. monocytogenes* DMST 31802	DMST
**NON*****-Listeria monocytogenes Listeria*** **SPECIES** (***n*** = **3**)	
*L. innocua* DMST 9011	DMST
*L. ivanovii* DMST 9012	DMST
*L. welshimeri* DMST 20559	DMST
**NON-*****Listeria*** **BACTERIAL STRAINS** (***n*** = **27**)	
***Salmonella*** **serovar** (***n*** = **10**)	
*S*. Typhimurium	DMST
*S*. Enteritidis	DMST
*S*. Stanley	DMST
*S*. Agona	DMST
*S*. Rissen	DMST
*S*. Anatum	DMST
*S*. Kedougou	DMST
*S*. Paratyphi A	DMST
*S*. Weltevreden	DMST
*S*. Typhi	DMST
***Shigella species*** (***n*** = **4**)	
*S. boydii*	DMST
*S. flexeri*	DMST
*S. sonnei*	DMST
*S. dysenteriae*	DMST
***Campylobacter*** **species** (***n*** = **4**)	
*C. jejuni* ATCC 33291	DMST
*C. coli* NCTC 11353	DMST
*C. lari* ATCC 43675	DMST
*C. fetus* ATCC 27374	DMST
*Escherichia coli* ATCC 25922	DPSWU
*Bacillus cereus*	DBSWU
*Staphylococcus aureus* ATCC 25923	DPSWU
*Pseudomonas aeruginosa* ATCC 27853	DPSWU
*Micrococcus luteus*	DBSWU
*Citrobacter diversus*	DBSWU
*Serratia marcescens*	DBSWU
*Enterobacter aerogenes*	DBSWU
*Klebsiella oxytoca*	DBSWU

*DMST, Department of Medical Sciences Culture Collection, National Institute of Health, Department of Medical Sciences, Tiwanon Road, Nonthaburi, 11000, Thailand; DPSWU, Department of Pathology, Faculty of Medicine, Srinakharinwirot University, HRH Princess Maha Chakri Sirindhorn Medical Center, Rangsit-Nakhonnayok Road, Ongkarak, Nakhon Nayok, 26120, Thailand; DBSWU, Department of Biology, Faculty of Science, Srinakharinwirot University, Sukhumvit Road, Bangkok, 10110, Thailand*.

A single colony of bacterial strain on brain heart infusion (BHI) agar (BBL, Becton Dickinson Microbiology Systems, Cockeysville, MD, USA) was cultured in BHI broth with constant shaking (250 rpm) at 37°C overnight. The extracted DNAs of all strains were obtained as previously described method (Wachiralurpan et al., 2017a). The rapid DNA extraction was performed by suspension of bacterial cell pellets in 100 μL of sterile deionized, nanopure water prior to boiling at 100°C for 10 min. The sterile deionized, nanopore water was used as negative control.

### DNA amplification

According to the previous report (Wachiralurpan et al., 2017a), a *plcB* primers set and DNA probe (Petty patent submission number 1601004756) were commercially synthesized by Bio Basic Inc., Canada. The LAMP and PCR reactions were performed according to the methods described in previous report (Wachiralurpan et al., 2017a). The negative controls were included in each DNA amplification run.

### Synthesis of the oligonucleotide probe-conjugated gold nanoparticles (GNPs)

Colloidal GNPs stabilized in citrate buffer with a particle diameter of 20 nm were commercially synthesized by Kestrel Bioscience Thailand Co. Ltd., Thailand. Conjugation reactions were conducted according to the procedure of Mirkin et al. (1996). Briefly, the thiol-modified DNA probe was immediately added to colloidal GNPs at a final concentration of 500 nM and incubated at 50°C with constant shaking (100 rpm) overnight in the dark. The solution was buffered by the addition of phosphate buffer (pH 7.5) to a final concentration of 10 mM containing 0.01% SDS and mixed gently for 10 s. Then, sodium chloride was added to a final concentration of 0.1 M and incubated for 48 h at 50°C with constant shaking (100 rpm) in the dark. After centrifugation at 15,000 rpm at 4°C for 30 min, the red oily precipitate was dispersed in wash buffer containing 10 mM phosphate buffer (pH 7.5), 0.01% SDS, and 0.1 M sodium chloride. The wash step was repeated twice to refine the precipitate. The concentration of GNPs was determined by measurement of the absorbance at the maximum wavelength (λ_max_) of 527 nm using a NanoDrop™ 2000 UV-vis Spectrophotometer (Thermo Scientific, USA) (Taton, 2002). The GNPs solution was stored at 4°C in the dark until use.

### Optimization of the GNP/DNA probe for LAMP product detection

A ratio of the optimum hybridization condition was modified from Suebsing et al. (2013). The hybridization for the detection of LAMP products was conducted in a total volume of 6 μL at 50°C for 10 min. Briefly, LAMP products were directly mixed with GNPs at a volume ratio of 3:3 μL. The effect of salt concentration-induced aggregation was subsequently tested at different final concentrations of MgSO_4_ (5–500 mM) to determine the optimum concentrations for the visual detection of color change and the annealing of the probe to its target. After adding MgSO_4_, the positive result appeared as the LAMP-GNP/DNA probe complexes were dispersed and the deep red color of the mixture could be observed by inspection by the naked eye, while the negative result was represented by aggregation of the GNP/DNA probe. The solution color changed from deep red to purple, then to colorless after the incubation period. The positive and negative results were determined at the wavelength of the maximum absorption (λ_max_) peak using a NanoDrop™ 2000 UV-vis Spectrophotometer (Thermo Scientific, USA).

### The detection limit and specificity of the LAMP-GNP/DNA probe assay

Under optimum conditions, a 10-fold serial dilution of pure culture and purified genomic DNA of *L. monocytogenes* DMST 17303 was determined as the last dilution of each positive test. Ten-fold serial dilutions were performed with sterile deionized nanopure water containing 400 ng μL^−1^ to 400 ag μL^−1^ and 2.82 × 10^8^ CFU mL^−1^ to 2.82 × 10^−1^ CFU mL^−1^, respectively.

The specificity of the LAMP-GNP/DNA probe assay was tested using five strains of *L. monocytogenes* and testing against DNA from other bacterial strains. A no-template reaction was employed as the negative control.

### Application on raw food samples

The ability of the assay to detect *L. monocytogenes* DNA was tested using 200 samples of raw chicken meat randomly collected from various open markets in Bangkok. The samples were then identified using the standard protocol recommended by the Bacteriological Analytical Manual (BAM) of the Food and Drug Administration (FDA) (Hitchins et al., 2016) at the Pathology Laboratory, Department of Pathology, Faculty of Medicine, Srinakharinwirot University, HRH Princess Maha Chakri Sirindhorn Medical Center, Thailand. One milliliter of the suspension of samples was extracted by using the rapid DNA extraction method as previously mentioned (Wachiralurpan et al., 2017a). The purified genomic DNA template was proceeded to LAMP assay.

The LAMP and LAMP-GNP/DNA probe assays were assessed by comparing the results with the FDA standard method. For any uncertain samples, the direct plating culture method was performed. Briefly, 1, 10, 100, and 500 μL of sample were plated individually on Oxoid Listeria Selective Agar (Oxford) (Basingstoke, UK) supplemented with Oxoid Listeria Selective Supplement (Oxford) (Basingstoke, UK) agar and incubated at 37°C for 72 h. Subsequently, the positive dark color colonies exhibiting esculin hydrolysis were isolated and subjected to Gram-staining, CAMP, catalase, sugar utilization, and motility tests.

## Results

### Optimization of LAMP and LAMP-GNP/DNA probe assays

**Figure 2:**
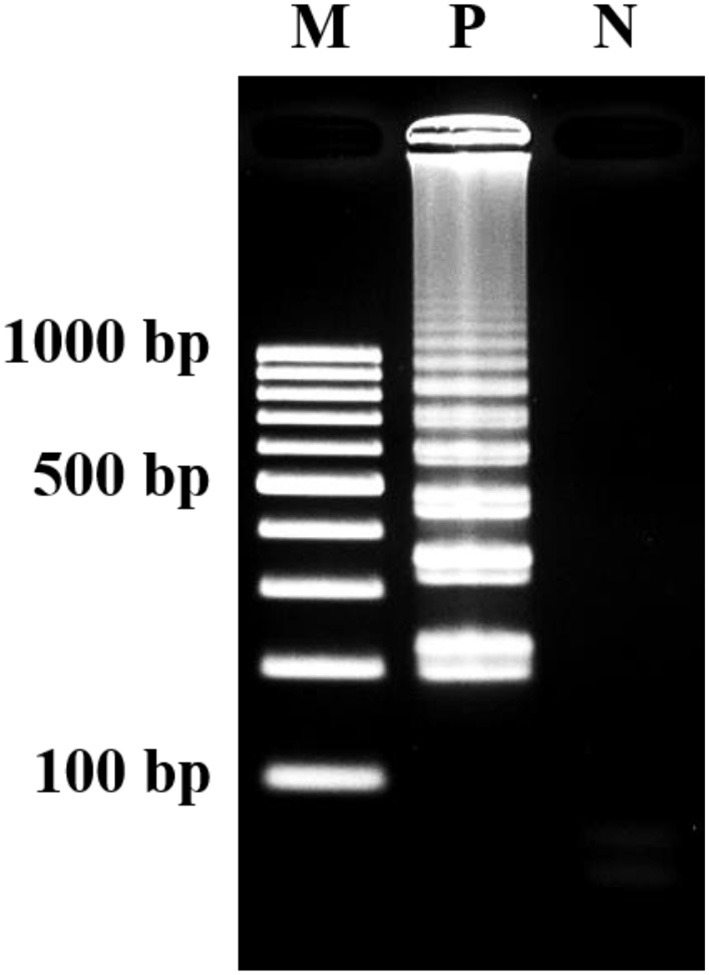
Visualization of LAMP products. Assessment was based on 2% agarose gel electrophoresis analysis. M, GeneRuler™ 100 bp DNA ladder marker (Thermo Scientific, USA); N, negative control (without DNA template); P, positive control (purified genomic DNA of *Listeria monocytogenes* DMST 17303).

The optimization of LAMP assay were conducted according to Wachiralurpan et al. (2017a). Analysis of LAMP products by 2% agarose gel electrophoresis (AGE) showed that a maximum ladder-like pattern was achieved at 63°C for 60 min (Figure 2).

**Figure 3:**
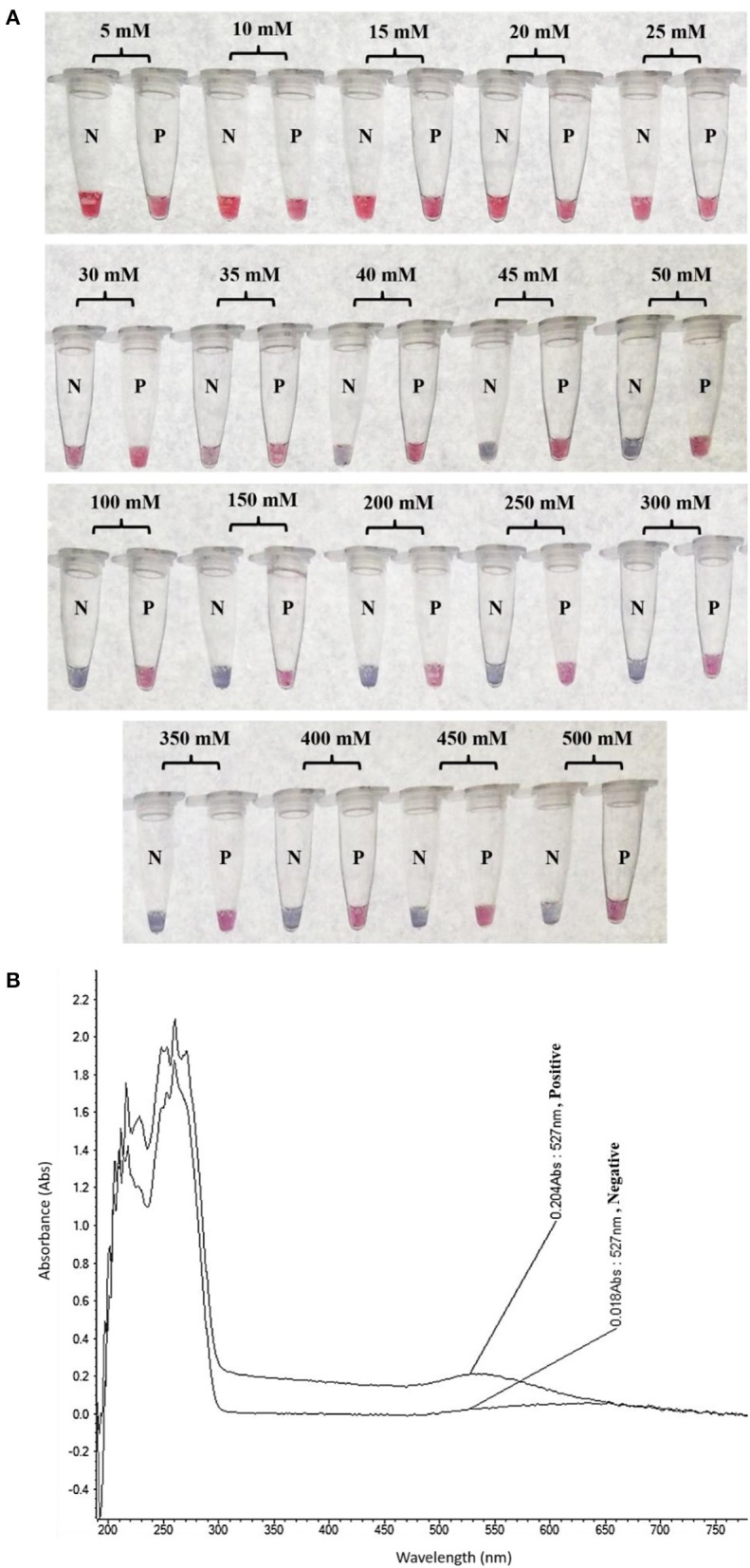
Optimization of LAMP-GNP/DNA Probe assay for colorimetric visual detection. Effects of the MgSO_4_ concentration on visualization of colorimetric detection **(A)**. UV-Vis spectra analysis corresponded to individual tube measured after adding 3 μL of 0.09 M MgSO_4_ (final conc— nM) into a mixture of LAMP product (3 μL) and GNP (3 μL) **(B)**. N, negative control (without DNA template); P, positive control (purified genomic DNA of *Listeria monocytogenes* DMST 17303).

The colorimetric detection of LAMP products (without heat denaturation) was achieved at the final concentration of 40–50 mM MgSO_4_. Concentrations of MgSO_4_ at 5–35 mM produced the false-positive pink color, while 100–500 mM resulted in the false-negative purple color (Figure 3A). The final concentration of 45 mM MgSO_4_ was confirmed by absorbance measurement at 527 nm (Figure 3B).

### Detection limit and specificity of LAMP-GNP/DNA probe assay

**Figure 4:**
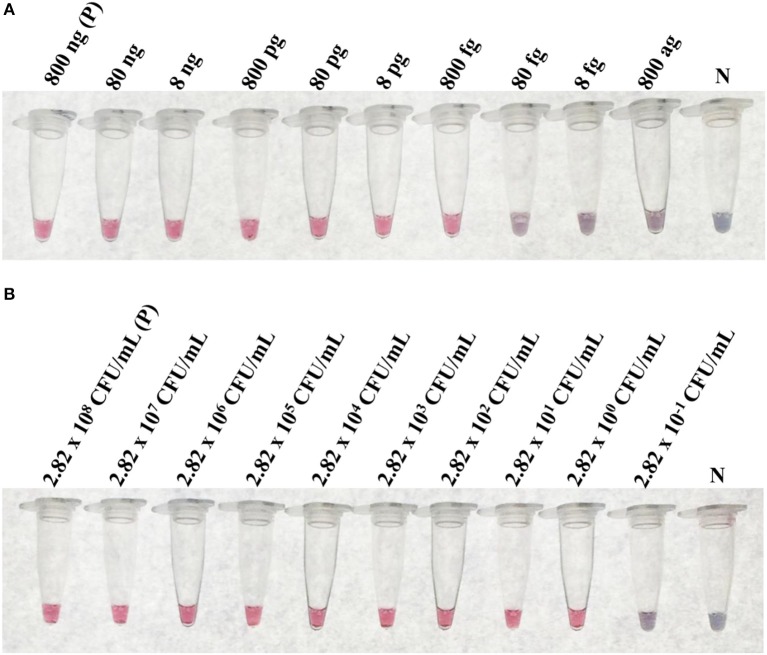
The limit of detection (LoD) of LAMP-GNP/DNA probe assay. The LoD on concentration of purified genomic DNA of *L. monocytogenes* DMST 17303 **(A)**. The LoD on CFU of pure culture of *L. monocytogenes* DMST 17303 **(B)**. N, negative control (without DNA template).

At the optimal conditions, the detection limit of the LAMP-GNP/DNA probe assay using purified *L. monocytogenes* DMST 17303 genomic DNA and bacterial cultures was 800 fg and 2.82 CFU mL^−1^, respectively (Figure 4).

**Figure 5:**
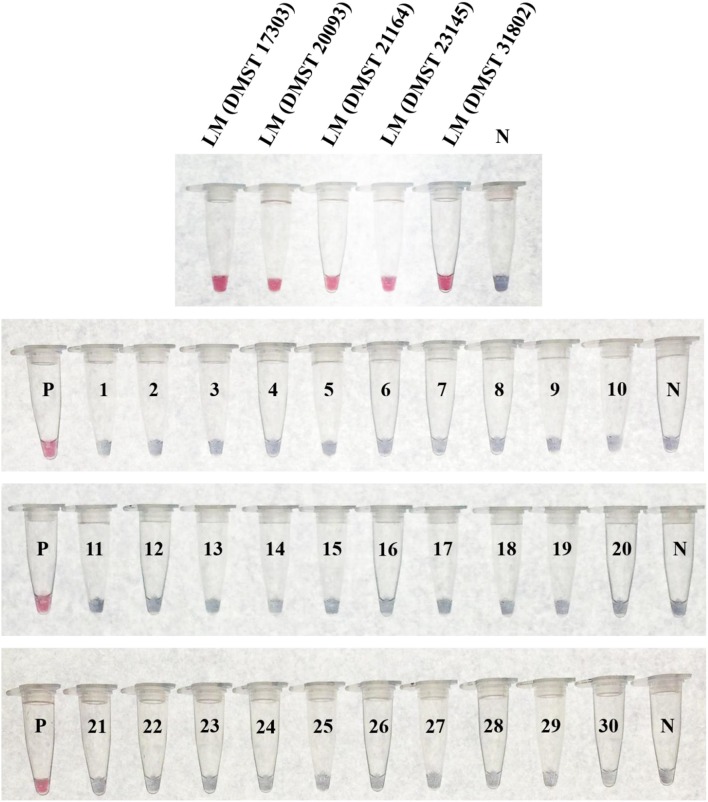
Specificity of LAMP-GNP/DNA probe assay against *Listeria monocytogenes*. A positive control of *L. monocytogenes* DNA template is indicated by “P.” A negative control of sterile deionized, nanopore water is indicated by “N.” LM, *Listeria monocytogenes*; 1, *Listeria innocua* DMST 9011; 2, *Listeria ivanovii* DMST 9012; 3, *Listeria welshimeri* DMST 20559; 4, *Salmonella* Typhimurium; 5, *Salmonella* Enteritidis; 6, *Salmonella* Stanley; 7, *Salmonella* Agona; 8, *Salmonella* Rissen; 9, *Salmonella* Anatum; 10, *Salmonella* Kedougou; 11, *Salmonella* Paratyphi A; 12, *Salmonella* Weltevreden; 13, *Salmonella* Typhi; 14, *Shigella boydii*; 15, *Shigella flexeri*; 16, *Shigella sonnei*; 17, *Shigella dysenteriae*; 18, *Campylobacter jejuni* ATCC 33291; 19, *Campylobacter coli* NCTC 11353; 20, *Campylobacter lari* ATCC 43675; 21, *Campylobacter fetus* ATCC 27374; 22, *Escherichia coli* ATCC 25922; 23, *Bacillus cereus*; 24, *Staphylococcus aureus* ATCC 25923; 25, *Pseudomonas aeruginosa* ATCC 27853; 26, *Micrococcus luteus*; 27, *Citrobacter diversus*; 28, *Serratia marcescens*; 29, *Enterobacter aerogenes*; 30, *Klebsiella oxytoca*.

The specificity testing of purified DNA from 35 bacterial isolates (Table 1) using the LAMP-GNP/DNA probe based on the *plcB* gene revealed that the tool was able to identify only the five *L. monocytogenes* isolates, without false positives (Figure 5). No cross reactions were observed with non-*L. monocytogenes Listeria* species or non-*Listeria* spp. as listed in Table 1.

### Application on raw food samples

The culture data from 200 raw chicken meat samples revealed that 51 and 149 samples were *L. monocytogenes* positive and negative, respectively. LAMP-AGE and LAMP-GNP/DNA probe assays demonstrated that 46 samples were *L. monocytogenes* positive, whereas PCR could detect only 36 positive samples (Table 2). Five culture-positive samples (No. 20, 40, 46, 104, and 150) were negative using the PCR, LAMP, and LAMP-GNP/DNA probe assays. After direct plating culture without enrichment in broth medium, all five isolates were confirmed as *L. monocytogenes*-negative samples.

**Table 2:** Comparison data of PCR, LAMP-agarose gel electrophoresis and LAMP-GNP/DNA probe for detection of *L. monocytogenes* in raw chicken meat samples.

**Diagnosis method**	**No. of positive results/Total^a^**	**Sensitivity (%)**	**Specificity (%)**	**Accuracy (%)**	**Total time of detection**
*plcB*-PCR	36/51	70.59	100	92.50	~2.30 h
*plcB*-LAMP-AGE	46/51	90.20	100	97.50	~2 h
*plcB*-LAMP-GNP/DNA probe	46/51	90.20	100	97.50	~1.10 h

a *Total number of positive results is based on testing by enrichment culture method (Hitchins et al., 2016)*.

The specificity, sensitivity, and accuracy of PCR, LAMP-AGE, and LAMP-GNP/DNA probe assays for the detection of *L. monocytogenes* were determined and compared to standard culture data. All tests exhibited 100% specificity. The sensitivity of the PCR, LAMP-AGE, and LAMP-GNP/DNA probe assays was 70.59, 90.20, and 90.20%, whereas the accuracy of each technique was 92.50, 97.50, and 97.50%, respectively (Table 2).

## Discussion

Previously, DNA detection assays based on species-specific genes of *L. monocytogenes*, such as *iap, hly, prfA*, and *plcA*, included LAMP, real-time LAMP turbidity, PCR and real-time PCR. In terms of efficiency, the PCR and real-time PCR assays could detect *L. monocytogenes* based on the listeriolysin O gene (*hly*) with a detection limit of 8–10 CFU (Rip and Gouws, 2009; Gianfranceschi et al., 2014). However, these assays required sophisticated equipment and post-amplification manipulations that took more time to obtain results. In the present study, an alternate approach has been employed: a novel GNP-based technique for bacterial pathogen detection (Mocan et al., 2017).

In this study, a rapid, specific, and sensitive colorimetric method, LAMP-GNP/DNA probe, has been established for the identification of *L. monocytogenes* based on the phosphatidylcholine-phospholipase C gene (*plcB*). The limit of detection was as low as 800 fg and 2.82 CFU mL^−1^ when purified genomic DNA and pure culture were tested, respectively. The technique was 10 times more sensitive than the conventional PCR assay (Wachiralurpan et al., 2017a). The specificity of the LAMP-GNP/DNA probe showed no cross-reaction with related bacterial species, which was equivalent to PCR-based methods.

By the PCR, LAMP, and LAMP-GNP/DNA probe assays, samples No. 20, 40, 46, 104, and 150 were false negatives, as the enrichment culture was *L. monocytogenes* positive. By direct culture on the plate without enrichment in broth medium, all were negative. It is possible that the contamination of *L. monocytogenes* in these raw chicken meat samples was <2.82 CFU mL^−1^, below the detection limit of the PCR, LAMP, and LAMP-GNP/DNA probe assays.

Concerning the simplicity and equipment independence, LAMP based assay such as LAMP-GNP/DNA probe can be easily and rapidly quantified by colorimetric visualization. This suggested the possibility of user-friendly on-site detection.

To avoid the side effects, the GNP/DNA probe was used for selective hybridization to target DNA without DNA-binding dye or a fluorescent probe and thermal cycler. After amplification, the assay required only 10 min for hybridization, performed using only a heating block or water bath. Then, the positive outcomes could be observed visually within 1 min after the addition of MgSO_4_.

In conclusion, the LAMP-GNP/DNA probe assay based on the *plcB* gene was suitable for use in the carrier screening diagnosis of *L. monocytogenes* in food products, providing a highly selective and cost-effective platform for the detection of DNA molecules. Considering the further spread of disease and economic impact, the LAMP-GNP/DNA probe has remarkable application in routine diagnostics as well as under field conditions and potential use in quarantine/surveillance programs. In addition, the techniques can be modified to use nanomaterial-assisted aptamer for optical sensing as aptasensors (Wang et al., 2010).

## Author contributions

SS, KC, SW, TS, SAr, SAu, and PS: Conceived and designed the experiments; SW: Performed the experiments; KC, SS, and SW: Analyzed the data; SW: Wrote the paper; KC: Revised and approved the final version of the paper.

### Conflict of interest statement

The authors declare that the research was conducted in the absence of any commercial or financial relationships that could be construed as a potential conflict of interest.
